# Kolaviron and L-Ascorbic Acid Attenuate Chlorambucil-Induced Testicular Oxidative Stress in Rats

**DOI:** 10.1155/2014/587015

**Published:** 2014-09-17

**Authors:** Ebenezer Tunde Olayinka, Ayokanmi Ore

**Affiliations:** Biochemistry Unit, Department of Chemical Sciences, Ajayi Crowther University, PMB 1066, Oyo, Oyo State, Nigeria

## Abstract

Chlorambucil (4-[4-[bis(2-chloroethyl)amino]phenyl]butanoic acid) is an alkylating agent, indicated in chronic lymphocytic leukaemia. Kolaviron (KV), a biflavonoid complex from* Garcinia kola,* and L-ascorbic acid (AA) are known to protect against oxidative damage* in vivo*. This study evaluates the protective capacity of KV and AA on chlorambucil-induced oxidative stress in the testes of rat. Twenty male Wistar rats (180–200 g) were randomized into four groups: I: control, II: chlorambucil (0.2 mg/kg b.w.), III: 0.2 mg/kg chlorambucil and 100 mg/kg KV, and IV: 0.2 mg/kg chlorambucil and 100 mg/kg AA. After 14 days of treatments, results indicated that chlorambucil caused significant reduction (*P* < 0.05) in testicular vitamin C and glutathione by 32% and 39%, respectively, relative to control. Similarly, activities of testicular GST, SOD, and CAT reduced significantly by 48%, 47%, and 49%, respectively, in chlorambucil-treated rats relative to control. Testicular MDA and activities of ALP, LDH, and ACP were increased significantly by 53%, 51%, 64%, and 70%, respectively, in the chlorambucil-treated rat. However, cotreatment with KV and AA offered protection and restored the levels of vitamin C, GSH, and MDA as well as SOD, CAT, GST, ACP, ALP, and LDH activities. Overall, kolaviron and L-ascorbic acid protected against chlorambucil-induced damage in the testes of the rat.

## 1. Introduction

The upsurge in the synthesis and use of anticancer agent has generated questions about the safety of these substances on normal tissues. Most drugs administered in cancer chemotherapy are well known to be associated with toxic side effects [1]. Among the most commonly reported toxic effects are those associated with organs such as the liver, kidneys, testes, and other functional parameters of the blood and immune system [2–6].

**Figure 1:**
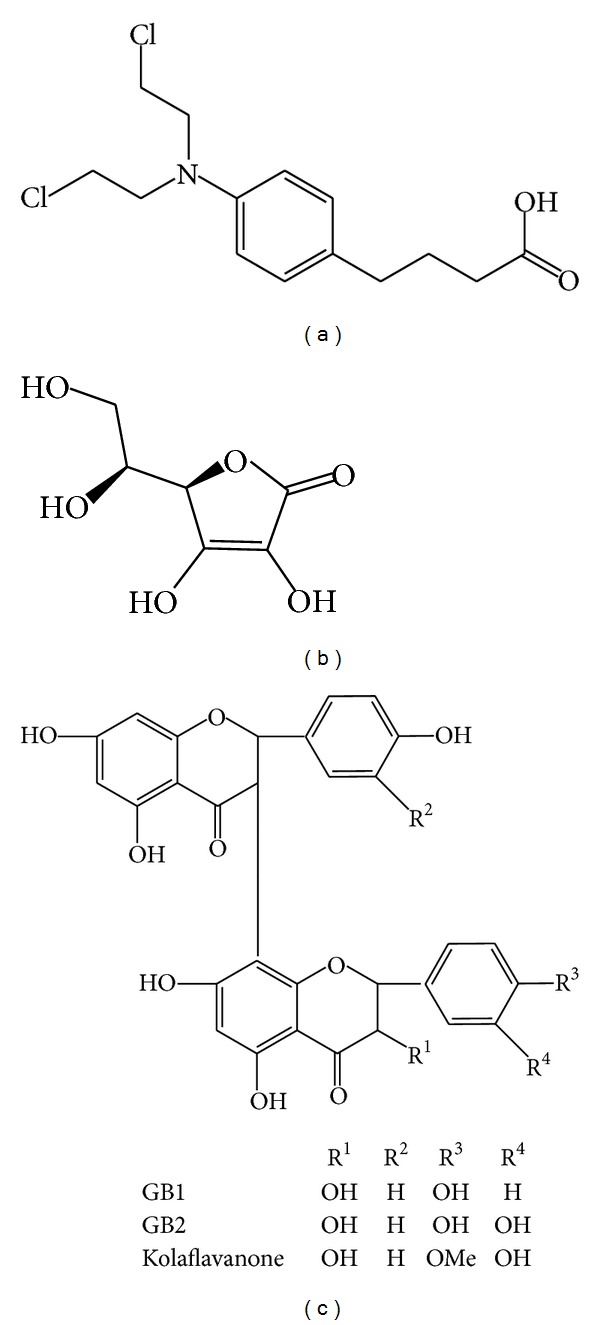
Chemical structure of (a) chlorambucil (4-[4-[bis(2-chloroethyl) amino] phenyl] butanoic acid), (b) L-ascorbic acid, and (c) kolaviron.

Chlorambucil (4-[4-[bis (2-chloroethyl) amino] phenyl] butanoic acid), Figure 1(a), is an orally available alkylating agent indicated in the treatment of chronic leukaemia [3, 7]. Chlorambucil is rapidly absorbed from the gastrointestinal tract and distributed to the liver, kidneys, and other organs. The metabolism of chlorambucil occurs predominantly in the liver by the hepatic drug metabolising enzymes leading to the formation of 3-(4-dehydrochlorambucil and phenylacetic acid mustard, 2-(4-(bis(2-chloroethyl)amino)phenyl)acetic acid) [8]. Phenylacetic acid mustard is further metabolized to inactive products which are excreted in the urine and faeces [9].

Chlorambucil and its derivatives form covalent bonds with proteins and DNA of neoplastic cells resulting in structural and functional damage to DNA [10]. While chlorambucil and its metabolites are cytotoxic to cancer cells, they may also be toxic to normal body cells. Toxic effects of chlorambucil in the form of haematological toxicity, hepatotoxicity, nephrotoxicity, neurotoxicity, and so forth have been reported [3, 4, 11–13]. Recent study from our laboratory suggested that the mechanism of toxicities elicited by chlorambucil may involve formation of free radicals or depletion of cellular antioxidant reserve [14].

The testes have evolved a formidable antioxidant defence to protect against the damaging effects of free radicals and oxidants which may have negative implication on spermatogenesis [15]. These comprise the nonenzymatic antioxidants including glutathione, ascorbic acid, and tocopherol and enzymatic antioxidants such as catalase, superoxide dismutase, glutathione peroxidase, glutathione reductase, and glutathione S-transferase. The testes are rich in lipid components and, therefore, exposure to high level of prooxidants may deplete the antioxidant reserve, thereby exposing the cell to excessive level of lipid peroxidation and ultimately tissue damage [16].

The natural antioxidant, kolaviron, is a fraction of the defatted ethanol extract of* Garcinia kola*, containing Garcinia biflavonoids GB1 and GB2 and kolaflavanone (Figure 1(c)). Kolaviron is of high safety profile and its antioxidant properties have been extensively studied* in vivo* [17]. Moreover, it is known to significantly prevent drug and chemical-induced organ toxicity and oxidative damage in experimental animal models [14, 18–20].

Many classes of antineoplastic agents are known to generate high levels of oxidative stress in biological systems [21], leading to tissue injuries. Injuries elicited by antineoplastic agents on vital organs have been reported in most cases but with few reports on reproductive organs, particularly the testes. Fewer reports are also available concerning chemotherapy-induced injuries due to generation of potent oxidants or disruptions of testicular antioxidant system by these agents. Besides, proper functioning of the testicular redox homeostasis is essential to efficient spermatogenesis and overall function of the testes. Chlorambucil has been reported in our previous study to induce oxidative stress in the liver of rats [14]. It is assumed that the administration of antioxidants along with chlorambucil may offer some protection against generation of reactive oxygen species (ROS) and oxidative damage to the testes. In addition, if the reactive species and oxidative stress caused by chlorambucil are responsible only for its toxic effects, antioxidants supplementation may reduce the severity of these toxic effects without interfering with the antineoplastic activity of the drug. Evidence suggests that administration of antioxidants along with chemotherapeutic agents has been found to effectively relieve toxic side effects [22–24]. In most cases, antioxidant has improved the cytotoxicity of antineoplastic agents. For instance, vitamin C is reported to enhance the antineoplastic activity of doxorubicin, cisplatin, and paclitaxil in human breast carcinoma cells [25]. Also, combination of vitamins A, C, and E improved the antitumor activity of doxorubicin in mice [26]. Several studies have also reported the potential of the plant derived flavonoids to inhibit the proliferation of cancer cells [27–30]. However, the present study was designed specifically to evaluate the protective role of kolaviron, a biflavonoid complex, and L-ascorbic acid on chlorambucil-induced oxidative stress in the testes of rats.

## 2. Materials and Methods

### 2.1. Chemicals and Assay Kits

Chlorambucil (Leukeran) was a product of Aspen Pharm Trading Limited, Dublin 1, Ireland. 1-Chloro-2,4-dinitrobenzene (CDNB), 5′,5′-dithiobis-2-nitrobenzoic acid (DTNB), glutathione (GSH), epinephrine, hydrogen peroxide (H_2_O_2_), and thiobarbituric acid (TBA) were all purchased from Sigma Chemical Company (London, UK). Assay kits for alkaline phosphatase (ALP) and acid phosphatase (ACP) were obtained from Randox Laboratories Ltd. (Antrim, UK). Assay kit for lactate dehydrogenase (LDH) was a product of Cypress Diagnostics, Belgium. All other reagents used were of analytical grade and were obtained from British Drug House (BDH), Poole, England.

### 2.2. Extraction of Kolaviron

Kolaviron was extracted from the fresh seeds of* Garcinia kola* (3.5 kg) and characterized according to the method of Iwu et al. [31]. Briefly, the powdered seeds were extracted with light petroleum ether (b.p. 40–60°C) in a Soxhlet extractor for 24 hr. The defatted, dried marc was repacked and then extracted with methanol. The extract was concentrated and diluted to twice its volume with distilled water and extracted with ethyl acetate (6.25 L). The concentrated ethyl acetate fraction yielded a golden yellow solid termed kolaviron which has been shown to consist of Garcinia biflavonoid GB-1 (3′′,4′,4′′′,5,5′′,7,7′′-heptahydroxy-3,8′′ biflavanone), GB-2 (3′′,4′,4′′′,5,5′′,5′′′,7,7′′-octahydroxy-3,8′′-biflavanone), and kolaflavanone (3′′,4′,4′′′,5,5′′,5′′′,7,7′′octahydroxy-4′′′-methoxy-3,8′′-biflavanone) (Figure 1). The purity and identity of kolaviron were determined by subjecting it to thin-layer chromatography (TLC) using Silica gel GF 254-coated plates and solvent mixture of methanol and chloroform in a ratio 1 : 4 v/v. The separation revealed the presence of three bands which were viewed under UV light at a wavelength of 254 nm with RF values of 0.48, 0.71, and 0.76 [32].

### 2.3. Animal Selection and Care

Twenty inbred male rats (Wistar strain) weighing between 180 and 200 g were obtained from the animal holding unit of the Department of Chemical Sciences, Ajayi Crowther University, Oyo, Nigeria. The rats were acclimatised under laboratory conditions prior to the commencement of the study. The animals were housed in wire meshed cages maintained at standard conditions of temperature and humidity with an alternating light cycle (12 hr light/dark). They were fed with standard rat pellet (Ladokun feeds, Ibadan, Nigeria) and were supplied water* ad libitum*. The experimental protocol relating to animal handling conformed to the Guidelines of the National Institute of Health—*Guide for the Care and Use of Laboratory Animals *(NIH Publication number 85-23 revised 1985: US Department of Health, Education and Welfare Bethesda, MA).

### 2.4. Drug Treatments

The rats were randomised into four experimental groups (I–IV) of 5 animals each. Group I (control) animals were administered distilled water. Animals in group II received chlorambucil at a dose of 0.2 mg/kg body weight (b.w.). Group III animals were coadministered chlorambucil (0.2 mg/kg b.w.) and kolaviron (100 mg/kg b.w.). Animals in group IV were coadministered chlorambucil (0.2 mg/kg b.w.) and L-ascorbic acid (100 mg/kg b.w.). The dose of chlorambucil administered to the animals was based on the recommended dose in the treatment of Hodgkin's lymphoma (0.1-0.2 mg/kg/day) [33], while doses of ascorbic acid and kolaviron were selected based on the results obtained from previous studies in experimental animals [34, 35]. Each of the drug doses was delivered orally in 1 mL solution, once daily using an oral cannula. The vehicle for chlorambucil and ascorbic acid was water while kolaviron was administered in corn oil. This has been included in the revised manuscript and highlighted. All the treatments lasted for a period of 14 days.

### 2.5. Animal Sacrifice and Collection of Testes

Animals were sacrificed 24 hours after the last treatments by cervical dislocation and the testes were carefully excised from each animal for preparation of cytosolic fraction and histopathological analysis. Testicular samples for histopathological analysis were immediately fixed in Bouin's solution for 24 hours.

### 2.6. Preparation of Cytosolic Fractions

The testes excised from rats were blotted of blood stains, rinsed in ice-cold 1.15% KCl, and homogenized in 4 volumes of ice-cold 0.01 M phosphate buffer (pH 7.4). The homogenates were centrifuged at 12,500 g for 15 min at −4°C (Eppendorf, UK) and the supernatants, termed the postmitochondrial fractions (PMF), were aliquoted and used for biochemical assays.

### 2.7. Determination of Testicular Protein Content

The protein concentration in the testicular homogenate was determined by the Biuret method of Gornall et al. [36] using bovine serum albumin as standard.

### 2.8. Assay of Testicular SOD, CAT, and GST

The method of Misra and Fridovich [37] as described by Magwere et al. [38] was used for the determination of testicular superoxide dismutase (SOD) activity by measuring the inhibition of autooxidation of epinephrine at pH 10.2 and 30°C. Testicular catalase activity was determined by the method of Asru [39] by measuring the reduction of dichromate in acetic acid to chromic acetate at 570 nm. Glutathione S-transferase (GST) activity was determined by the method described by Habig et al. [40] using 1-chloro-2,4-dinitrobenzene (CDNB) as substrate.

### 2.9. Assay of Testicular GSH, Ascorbic Acid, and Level of Lipid Peroxidation

Testicular reduced glutathione (GSH) level was determined according to the method of Jollow et al. [41]. The chromophoric product resulting from the reaction of Ellman's reagent with the reduced glutathione, 2-nitro-5-thiobenzoic acid, possesses a molar absorption at 412 nm which was measured in a spectrophotometer. Reduced GSH is proportional to the absorbance at 412 nm. The ascorbic acid concentration was determined according to the method of Jagota and Dani [42]. Ascorbic acid in testicular samples reacts with Folin's reagent, an oxidizing agent to give a blue color which has its maximum absorption at 760 nm. The extent of lipid peroxidation (LPO) was estimated by the method of Varshney and Kale [43]. The method involved the reaction between malondialdehyde (MDA) a product of lipid peroxidation and thiobarbituric acid to yield a stable pink chromophore with maximum absorption at 532 nm.

### 2.10. Assay of Testicular ACP, ALP, and LDH

Testicular acid phosphatase (ACP), alkaline phosphatase (ALP), and lactate dehydrogenase (LDH) activities were determined using Randox diagnostic kits. ACP and ALP activities were determined by the method of Tietz [44]. The p-nitrophenol formed by the hydrolysis of p-Nitrophenyl phosphate confers yellowish colour on the reaction mixture and its intensity can be monitored at 405 nm to give a measure of enzyme activity. Determination of testicular LDH activity was based on the method of Cabaud and Wroblewski [45].

### 2.11. Testicular Histology

The method of Baker and Silverton [46] was employed for the processing of the testicular samples for histopathological studies. Fixed testicular samples were dehydrated in graded ethanol and embedded in paraffin wax. A thin section was made from each testicular tissue and was stained with hematoxylin and eosin, followed by examination under a light microscope.

### 2.12. Statistical Analysis

The results were expressed as mean of 5 replicates ± SD. Data obtained were subjected to one-way analysis of variance (ANOVA) and complemented with Duncan's multiple range test using SigmaPlot Statistical Software. A value of *P* < 0.05 was accepted as statistically significant.

## 3. Results

### 3.1. Protective Effect of Kolaviron and L-Ascorbic Acid on Chlorambucil-Induced Changes in Testicular SOD, CAT, and GST Activities in Rats

Following two-week exposure to chlorambucil, the testicular activities of SOD, CAT, and GST were significantly decreased by 47%, 49%, and 48%, respectively, in chlorambucil-treated animals in comparison with the controls (Table 1). Kolaviron and ascorbic acid cosupplementation with chlorambucil significantly attenuated the activities of these testicular antioxidant enzymes toward the control levels.

### 3.2. Protective Effect of Kolaviron and L-Ascorbic Acid on Chlorambucil-Induced Changes in Testicular Level of GSH, Ascorbic Acid, and MDA in Rats

**Figure 2:**
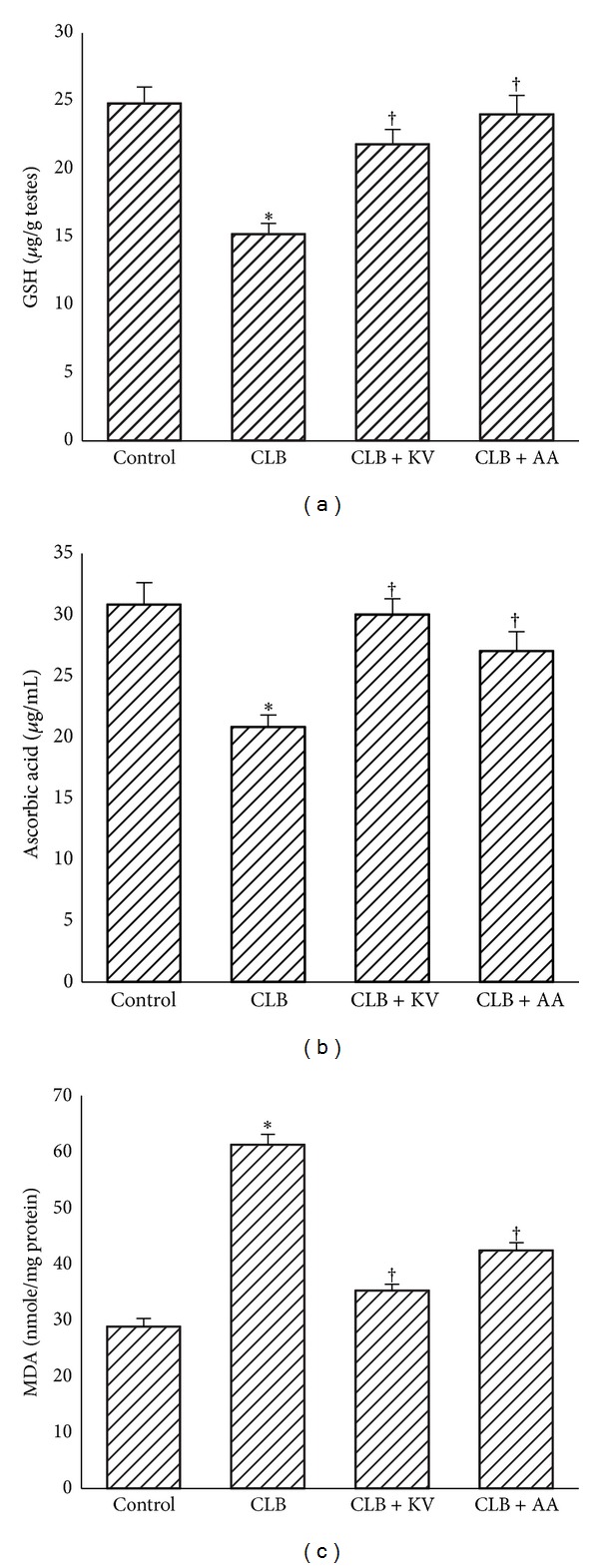
Protective effect of kolaviron and L-ascorbic acid on chlorambucil-induced changes in testicular level of GSH, ascorbic acid, and MDA in rats. Results are expressed as mean ± S.D (*n* = 5); GSH: reduced glutathione, MDA: malondialdehyde, CLB: chlorambucil (0.2 mg/Kg b.w.), KV: kolaviron (100 mg/Kg b.w.), and AA: ascorbic acid (100 mg/Kg b.w.). ∗Significantly different from CTRL; ^†^Significantly different from CLB.

As presented in Figure 2, a significant decrease by 39% and 32% in testicular reduced glutathione and vitamin C (nonenzymic antioxidants) was observed following chlorambucil exposure for two weeks with a corresponding significantly elevated testicular MDA content (a product of lipid peroxidation). However, coadministration of chlorambucil with kolaviron and vitamin C acid, respectively, significantly ameliorated the decrease in testicular GSH, vitamin C and increase in MDA (Figure 2).

### 3.3. Protective Effect of Kolaviron and L-Ascorbic Acid on Chlorambucil-Induced Changes in Activities of Testicular ACP, ALP, and LDH

**Figure 3:**
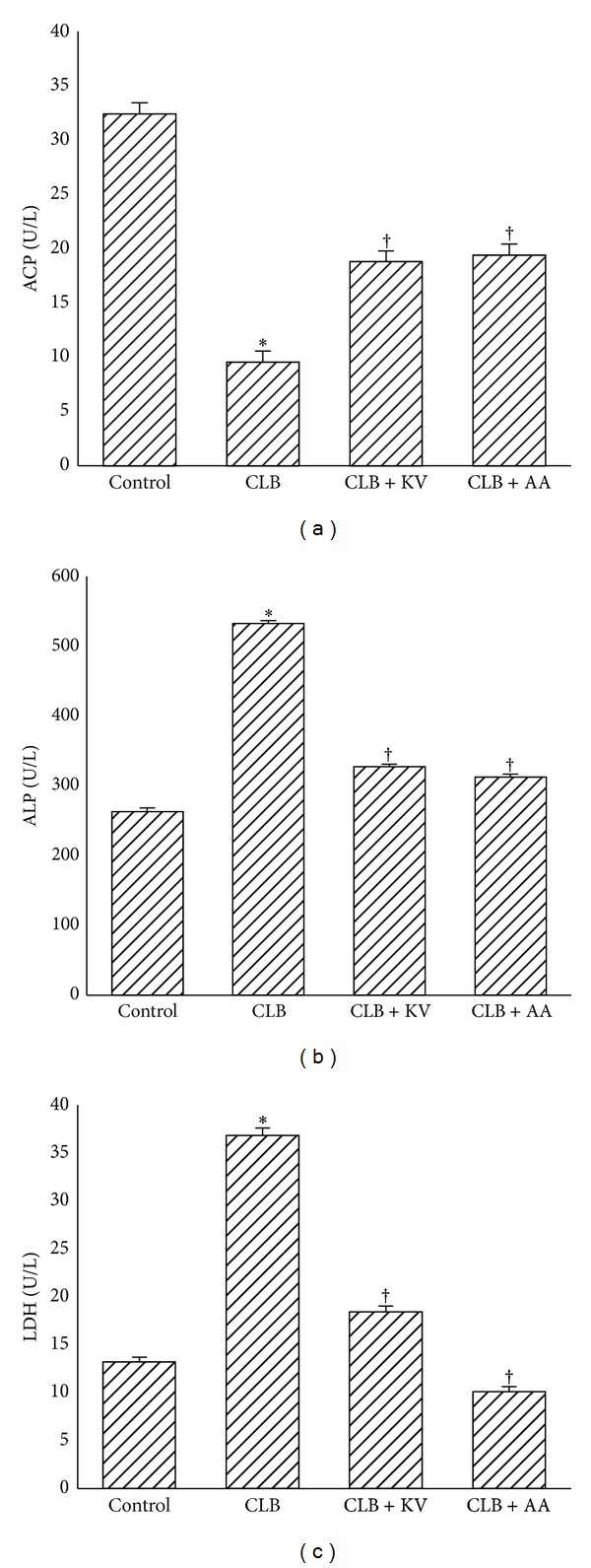
Protective effect of kolaviron and L-ascorbic acid on chlorambucil-induced changes in testicular ACP, ALP, and LDH in rats. Results are expressed as mean ± S.D (*n* = 5); ACP: acid phosphatase, ALP: alkaline phosphatase, LDH: lactate dehydrogenase, CLB: chlorambucil (0.2 mg/Kg b.w.), KV: kolaviron (100 mg/Kg b.w.), and AA: ascorbic acid (100 mg/Kg b.w.). ∗Significantly different from CTRL; ^†^Significantly different from CLB.

The testicular acid phosphatase (ACP) activity (Figure 3(a)) was significantly reduced by 70% while alkaline phosphatase (ALP) and lactate dehydrogenase (LDH) activities showed a significant increase by 59% and 64%, respectively, in the chlorambucil-treated animals with respect to the control (Figures 3(a), 3(b), and 3(c)). Kolaviron or ascorbic acid cosupplementation with chlorambucil significantly restored the activities of these testicular enzymes toward control.

### 3.4. Protective Role of Kolaviron and L-Ascorbic Acid against Chlorambucil-Induced Damage to Testicular Histology

**Figure 4:**
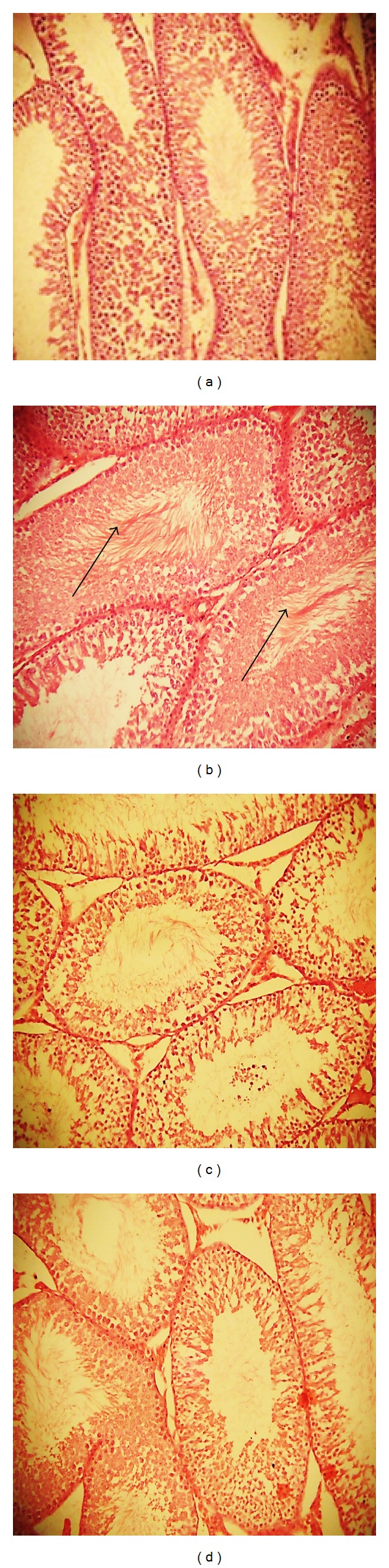
Photomicrograph showing protective role of kolaviron and L-ascorbic acid on chlorambucil-induced damage on testicular morphology (hematoxylin and eosin stain). (a) Control: no visible lesions seen. (b) Chlorambucil group: there are immature germinal cells in the lumen. (c) Chlorambucil + kolaviron group: no visible lesions seen; (d) chlorambucil + ascorbic acid group: no visible lesions seen.

Representative photomicrographs of the testicular sections from rats are shown in Figure 4. The histological study of the testicular section of the control rats showed a typical normal cellular architecture with no lesion. However, chlorambucil exposure for two weeks resulted in formation of immature germinal cells in the lumen (Figure 4(b)). However, coadministration of chlorambucil with kolaviron and ascorbic acid restored the testicular histoarchitecture with distinct cellular arrangements as observed for control animals.

## 4. Discussion

This study investigated the protective role of orally administered kolaviron and ascorbic acid in chlorambucil-induced testicular toxicity in rat. Chlorambucil is an anticancer agent indicated for chronic leukaemia and may have the capacity to induce oxidative stress or deplete tissue antioxidant reserve during chemotherapy [14]. The testis is an organ involved in spermatogenesis and is normally exposed to a low oxygen level. Despite the low oxygen level, the testes are known to be vulnerable to oxidative stress and peroxidative damage, due to the abundance of highly unsaturated fatty acids [15]. Testicular oxidative stress plays an important role in conditions known to be detrimental to male fertility. These conditions include exposure to certain drugs and environmental toxicants. Series of studies on antineoplastic agents have shown that they can cause oxidative stress in the testes with resulting disturbance in testicular functions [6, 47]. However, the testes possess formidable antioxidant defence systems comprising both enzymatic and nonenzymatic antioxidants. The enzymatic constituents of this defence system are made up of catalase (CAT), superoxide dismutase (SOD), glutathione S-transferase (GST), and glutathione peroxidase (GPx). The nonenzymatic constituents include the ascorbic acid (AA), reduced glutathione (GSH), and vitamin E.

In this study, we observed a significant reduction in testicular activities of superoxide dismutase (SOD), catalase (CAT), and glutathione S-transferase as a result of CLB administration. SOD catalyzes the reaction involving a rapid dismutation of superoxide radical to hydrogen peroxide and dioxygen while CAT converts the hydrogen peroxide formed in this process and other cellular processes into water and molecular oxygen. Reduction in the activities of SOD and CAT by chlorambucil may predispose the testes to oxidative damage [47]. Glutathione S-transferase (GST), on the other hand, is an enzyme involved in the detoxification of ingested xenobiotics in the liver. GST activity is also present in other tissues including the testes where it functions as part of the antioxidant defense mechanism that scavenge and suppress the formation of ROS [48]. In addition to the formation of glutathione S-conjugates of drugs, GST also possesses antioxidant capabilities. It catalyses the reduction of peroxide-containing compounds in the cell and this peroxidase activity exhibited by GST is however dependent on availability of GSH [48]. Coadministration of kolaviron and L-ascorbic acid offers protection against oxidative stress in the testes of the animal by increasing the activities of antioxidant enzymes in the experimental animals.

AA is a vital antioxidant of the aqueous phase of the cell and rapidly scavenges free radicals. It also plays an important role in the regeneration of vitamin E—the membrane bound antioxidant [49]. The AA in the testes is maintained in a reduced state by GSH dependent dehydroascorbate reductase in the testes. Suppression of testicular AA and GSH following administration of antineoplastic drugs has been reported in several studies [50, 51]. The observed reduction may be caused by the presence of free radicals generated during the metabolism of the drug, resulting in the consumption of the available AA and GSH in the testes. Reduction in testicular activities of enzymatic and levels of nonenzymatic antioxidants can predispose the testes to excessive oxidative stress. Suppression of testicular antioxidant system has been widely associated with oxidative stress. Uncontrolled oxidative stress may result in membrane lipid peroxidation and, ultimately, testicular damage and loss of testicular functions. The decrease in the activities of SOD, CAT, and GST in CLB-exposed rat testicular cells may have increased the observed level of lipid peroxidation. This observation is consistent with previous reports [52–54]. The observed reduction in testicular lipid peroxidation may be related to the protection of the antioxidant defense system in the rats by kolaviron and AA [55].

Activities of testicular marker enzymes such as acid phosphate (ACP), alkaline phosphate (ALP), and lactate dehydrogenase (LDH) are considered functional indicators of spermatogenesis. The reduction in the activity of ACP by the administration of CLB was supported by Ananthan and Kumaran [56] where administration of Mancozeb (a fungicide of ethylene-bis-dithiocarbamate group) significantly decreased the activity of ACP in the testis of rat. Spermatogenic cells contain acid phosphatase and the specific activity of this enzyme increases as the germ cells differentiate from spermatogonia into spermatocytes and spermatids [57]. Lysosomal acid phosphatases also participate in the intracellular digestion of endogenous and phagocytosed exogenous compounds containing phosphate residues. In the acrosome, they may be involved in the penetration of the spermatozoon through the egg [57]. The increase in the activity of ACP in the testes of animals administered KV and AA may be related to increased lysosomal activity in the testes. ALP and LDH play an important role in the process of spermatogenesis and have been shown to be vital for sperm survival and motility [58]. The increased activity of testicular ALP and LDH observed in this study may reflect testicular degeneration [53]. LDH is closely associated with spermatogenesis and testicular development. Increase in the activity of this enzyme in the chlorambucil-treated rats may also be resulting from adaptation to improve spermatogenesis and testicular development from oxidative damage [59]. KV and AA effectively attenuated the activities of ACP, ALP, and LDH.

The degeneration of testicular histological structure by CLB may be a result of oxidative damage. This can also result in the degeneration of germ cells and poor sperm quality [60]. KV and AA supplementation prevented the degeneration of germ cells as evidenced by histological evaluation. Potential evidences from the literature, supporting the role of ROS-mediated oxidative stress, may be responsible for the mechanism of chlorambucil-induced testicular toxicity observed [14]. The observed attenuation properties of KV and AA in this study may be due to their antioxidant properties [23, 61], which may be involved in the scavenging of radical species generated by CLB.

## 5. Conclusion

From our present study, it may be suggested that chlorambucil impairs testicular antioxidant system and causes degenerative changes in the germ cells. However, kolaviron and ascorbic acid positively modulate the effect of the drug on the antioxidant status and effectively prevent chlorambucil-induced testicular toxicity.

## Figures and Tables

**Table 1:** Protective effect of kolaviron and L-ascorbic acid on chlorambucil-induced changes in testicular SOD, CAT, and GST activities in rats.

Treatment groups	SOD (units)	CAT (*μ*moles/min/mg protein)	GST (nmole/min/mg protein)
Control	38.6 ± 1.9	8.14 ± 0.1	18.8 ± 1.2
CLB	20.4 ± 1.8*	4.12 ± 0.3*	9.74 ± 0.8*
CLB + KV	34.0 ± 1.6^∗†^	7.14 ± 0.2^∗†^	17.14 ± 0.9^∗†^
CLB + AA	32.8 ± 2.5^∗†^	7.60 ± 0.4^∗†^	16.0 ± 1.1^∗†^

Results are expressed as mean ± S.D (*n* = 5); SOD: superoxide dismutase, CAT: catalase, GST: glutathione S-transferase, CLB: chlorambucil (0.2 mg/Kg b.w.), KV: kolaviron (100 mg/Kg b.w.), and AA: ascorbic acid (100 mg/Kg b.w.). ∗Significantly different from control; ^†^Significantly different from CLB.
